# Impact of Semi-Permanent Nail Polish on Forensic DNA Profiling and Phenotyping from Fingernails

**DOI:** 10.3390/genes17030322

**Published:** 2026-03-16

**Authors:** Giulia Fazio, Sara Amurri, Arianna Giorgetti, Filomena Melchionda, Chiara Turchi, Susi Pelotti, Carla Bini

**Affiliations:** 1 Department of Medical and Surgical Sciences, Section of Legal Medicine, University of Bologna, 40126 Bologna, Italy; giulia.fazio2@unibo.it (G.F.); sara.amurri4@unibo.it (S.A.); arianna.giorgetti@unibo.it (A.G.); carla.bini@unibo.it (C.B.); 2 Department of Biomedical Sciences and Public Health, Marche Polytechnic University, 60126 Ancona, Italy; f.melchionda@staff.univpm.it (F.M.); c.turchi@staff.univpm.it (C.T.)

**Keywords:** DNA profiling, fingernails, DNA recovery, semi-permanent nail polish, forensic DNA phenotyping

## Abstract

Background/Objectives: The increasing global trend in nail beautification may lead to analyses of nails with semi-permanent polish for the identification of degraded human remains. This study aimed to evaluate the effects of cosmetic nail treatment on forensic STR DNA profiling and phenotyping of eye, hair, and skin colour characteristics using a massively parallel sequencing (MPS) assay. Methods: Forty-two nail samples obtained from 21 volunteers, classified in “new”, occasional and regular semi-permanent polish users, were submitted to DNA analysis. Results: The use of semi-permanent nail polish, particularly when applied repeatedly, resulted in a significant reduction in DNA recovery, but it did not affect STR typing for personal identification. Mixed STR profiles were observed in 28.6% of the samples, indicating that the nail washing procedure employed before DNA extraction did not completely remove the foreign DNA; however, this could be useful depending on the forensic context. FDP analysis was successfully applied on nails with semi-permanent polish that showed a good quantity of DNA and single-source profiles. Conclusions: The results highlight the evidentiary value of fingernails even if treated with semi-permanent nail polish that should still be regarded as a source of DNA for personal identification and further investigation in the forensic context.

## 1. Introduction

Highly degraded, fragmented, and/or poorly preserved human remains represent a frequent challenge in forensic investigations, including violent crimes, mass disaster scenarios, and kinship investigations requiring the exhumation of human remains. In these contexts, DNA profiling based on Short Tandem Repeats (STRs) and Single-Nucleotide Polymorphisms (SNPs) represents the standard for human identification [1,2,3,4]. Additional investigative analysis, such as forensic DNA phenotyping (FDP), can provide predictive information on externally visible characteristics (EVCs), biological age, and biogeographical ancestry [5]. Similarly, the applicability of FDP analysis may be constrained by limitations relating to the quality and quantity of the available DNA [6,7].

Bones and teeth are usually considered the tissues of choice for DNA analysis for degraded human remains, although DNA isolation from these sources can be challenging and time-consuming [8]. Where soft tissues are limited or degraded, previous studies have proposed the use of nail clippings as an alternative source of DNA for genetic human identification, due to their composition, resistance to decay and easy collection and storage [9,10,11].

In recent years, nail beautification using long-lasting nail polish (semi-permanent polish) has gained worldwide popularity and is used by millions of individuals for its low cost, persistency, and easy application, including in domestic setting [12]. Unlike traditional nail polish, the most frequently listed compounds on the labels are acrylate, cyanoacrylate and methacrylate monomer, which require ultraviolet light (UV) for polymerisation and hardening [13]. Both the application and removal processes expose the nails to chemical and mechanical stress, due to the use of aggressive solvents such as acetone and manual removal of the product [14,15]. Consequently, the amount of DNA available for forensic human identification may be reduced and degraded [16,17]. Furthermore, the presence of foreign DNA encapsulated in cosmetic gel nails may further complicate the genetic interpretation of STR markers for human identification purposes [18].

The present study investigates the effects of semi-permanent nail polish treatment on DNA quantity and quality, STR profiling, and the prediction of eye, hair, and skin colour using FDP analysis.

## 2. Materials and Methods

### 2.1. Experimental Design and Sample Collection

This study was conducted in accordance with ethical standards and was approved by the Bioethical Committee of the University of Bologna (Protocol no. 0175936). Twenty-one female volunteers, aged between 25 and 50 years, were included in the study. Written informed consent was obtained from all volunteers, who were asked to complete a questionnaire collecting phenotypic information on eye, hair, and skin colour, and photographic data were collected. Participants were invited to select the categories that best represented their physical appearance, following criteria reported in the literature, with three categories for eye colour, four for hair colour, and five for skin colour [19].

**Figure 1:**
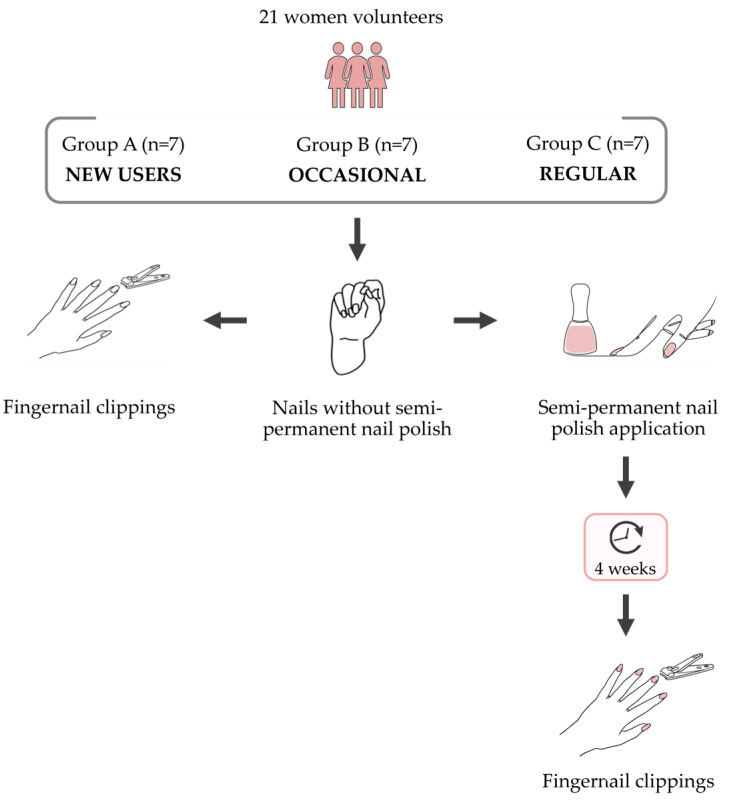
Schematic representation of the experimental design and timing of fingernail sample collection.

The participants were divided into three groups according to the frequency of semi-permanent nail polish use. The first group comprised new users, defined as individuals who had never used semi-permanent nail polish prior to enrolment in the study (Group A, *n* = 7). The second group comprised occasional users, defined as individuals who used semi-permanent nail polish intermittently (4–5 applications per year) (Group B, *n* = 7), and the third group comprised regular users, defined as individuals who regularly had semi-permanent nail polish applied (approximately 1 application every 20–30 days) (Group C, *n* = 7) (Figure 1).

**Figure 2:**
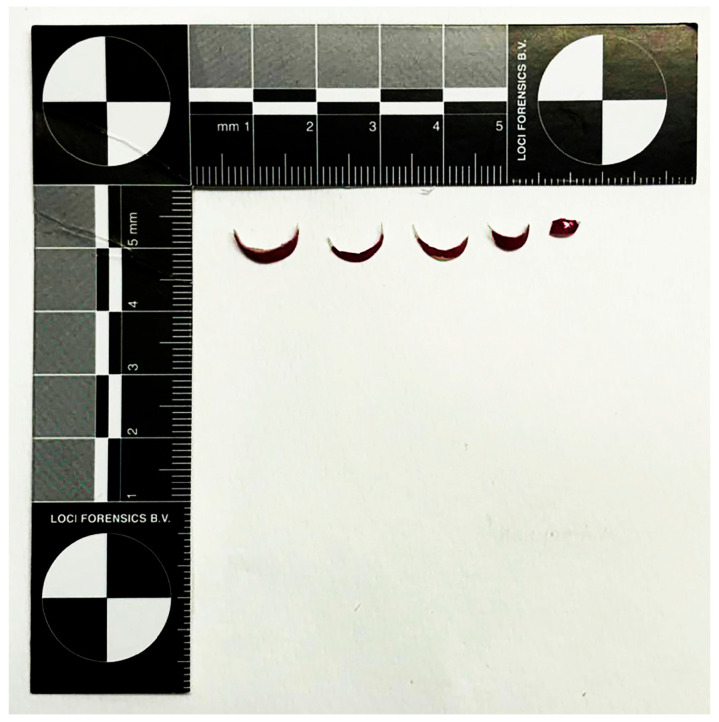
Actual photograph of fingernails clipped in our study and measured with a millimetre scale.

Fingernail clippings were collected from each volunteer before and four weeks after the application of semi-permanent nail polish, resulting in a total of 42 samples (Figure 1 and Figure 2). Semi-permanent nail polish was applied to the nails of each volunteer following a standardised procedure.

First, each female volunteer washed her hands with water and soap and dried them with a clean paper towel. Then, each nail was filed using a 240-grit buffer to gently remove surface shine and standardise the nail surface. Nail prep was then applied to further dehydrate the nail plate, followed by the application of an acid-free primer, which was allowed to air-dry for 30 s. A thin layer of base gel defence coat was subsequently applied and cured under a 48 W UV-LED lamp. Two thin layers of coloured gel polish were then applied sequentially, with each layer being individually cured. Finally, the top coat was applied and cured to seal the system. The curing times for each product and product’s ingredients are reported in Table 1. Photocuring was performed after the application of each individual layer.

An aliquot of the cosmetic products, collected as a negative control, was deposited on a previously sterilised microscope slide and allowed to dry following the same protocol applied to the fingernails. Then, the aliquot was powdered using a sterile cutter and transferred into a sterile tube for DNA extraction. Reference biological material from the volunteers was collected using sterile buccal swabs (Copan Italia S.p.A., Brescia, Italy). After DNA collection, the fingernails and swabs were stored at −20 °C for 30 days to maintain sample integrity pending the full nail set being collected for batch processing.

### 2.2. DNA Purification and Quantification

Nails without and with semi-permanent nail polish were weighed, and, respectively, 10 mg and 12 mg were submitted to DNA extraction, considering 2 mg the weight of the applied cosmetic products, experimentally determined. Fingernail clippings were twice washed first in 500 μL distilled water at 50 °C for 15 min and then in 500 μL absolute ethanol [11] to remove potential exogenous DNA not originating from the nail matrix and subsequently air-dried for 10 min. DNA purification from all collected samples was performed using the Maxwell^®^ FSC DNA IQ™ Casework Kit (Promega, Madison, WI, USA) on a Maxwell^®^ RSC Instrument, following the manufacturer’s protocol, “Automated Purification of DNA from Fingernail Clippings”. The aliquot collected from the semi-permanent nail polish used in this study was also subjected to DNA extraction, using the same protocol. Reference buccal swabs were processed using ReadyAMP™ Genomic DNA Purification System Kit (Promega, Madison, WI, USA). A negative extraction control was included in each extraction session. Quality, quantity and presence of inhibition of the isolated DNA were assessed using the PowerQuant System (Promega, Madison, WI, USA) on the QuantStudio 5 Real-Time PCR System for Human Identification (Thermo Fisher Scientific, Waltham, MA, USA), in a reduced volume of 10 µL, with 2 μL of DNA extract added (a modification that was previously validated in our laboratory). The raw data was analysed by the PowerQuant Analysis Tool (Promega, Madison, WI, USA).

### 2.3. DNA Profiling and DNA Phenotyping Analysis

DNA profiling of all fingernails samples was carried out by amplifying 0.3 ng as the total amount of genomic DNA template using the GlobalFiler™ IQC PCR Amplification Kit (Thermo Fisher Scientific, Waltham, MA, USA), which amplifies 21 autosomal STRs, 1 Y-STR (DYS391), 1 insertion/deletion polymorphic marker on the Y chromosome (Y indel), the amelogenin locus and 2 Internal Quality Control (IQC) markers, on a VeritiPro™ Thermal Cycler 96-well instrument (Thermo Fisher Scientific, Waltham, MA, USA), in a total volume of 5 µL. Positive and negative controls were included. The amplified DNA was subsequently analysed on a SeqStudio Genetic Analyzer (Thermo Fisher Scientific, Waltham, MA, USA) and interpreted using GeneMapper^®^ ID-X v1.6 (Thermo Fisher Scientific, Waltham, MA, USA), with an analytical threshold of 100 relative fluorescence units (RFU).

FDP analysis was performed on the 6 samples with semi-permanent nail polish yielding at least 0.5 ng/μL of DNA, characterised by a complete single-source genetic profile matching the corresponding volunteer. Analysis was performed using an MPS assay comprising 41 SNPs from the HIrisPlex-S system [19], according to Melchionda et al. [7]. The assay was designed using the Ion AmpliSeq Designer web tool (Thermo Fisher Scientific, Waltham, MA, USA, https://ampliseq.com/, accessed on 7 March 2019), with the FFPE (formalin-fixed paraffin-embedded) DNA type option selected so that the amplicons were less than 180 bp in length, allowing the analysis of degraded samples. MPS libraries were manually prepared in half-reaction volume using the Precision ID Library Kit (Thermo Fisher Scientific, Waltham, MA, USA) following the manufacturer’s protocols (MAN0017767, rev C.0). Amplifications were performed with 0.4–1 ng of DNA input using 23 PCR cycles. After partial primer digestion and ligation steps, libraries were purified with Agencourt™ AMPure™ XP Reagent (Beckman Coulter, Brea, CA, USA), quantified using the Ion Library TaqMan^®^ Quantification Kit (Thermo Fisher Scientific, Waltham, MA, USA), and diluted to a final concentration of 40 pM. Emulsion PCR and chip loading were performed using the Ion Chef™ Instrument (Thermo Fisher Scientific, Waltham, MA, USA) and sequencing was carried out on the Ion Gene Studio™ S5 System using an Ion 520™ Chip (Thermo Fisher Scientific, Waltham, MA, USA). All raw data were processed using Torrent Suite software (v. 5.12.3), and sequencing reads were aligned to the human reference genome (GRCh37/hg19). Genotype calls were performed using the HID SNP Genotyper Plugin (v. 4.3.2) with default parameters. A secondary analysis was carried out using Integrative Genomics Viewer (IGV, v. 2.8.0; Broad Institute and UC San Diego) [20] to inspect ambiguous calls that had been flagged during quality control. For final interpretation, the locus call threshold was set at 50 reads, with a minimum allele frequency of 0.1 for heterozygote calling.

### 2.4. Data Interpretation and Statistical Analysis

Quantification data, including autosomal and Y chromosome DNA quantity and degradation index, were analysed using descriptive statistics and are reported as mean and standard deviation (SD) and as median and interquartile range (IQR). A non-parametric paired *t*-test Wilcoxon matched-pair signed rank test was used to compare DNA amounts, considering both autosomal and Y chromosome DNA, between samples without and with semi-permanent nail polish; the same analysis was applied to the degradation index.

DNA quantity and quality were compared among Groups A, B, and C, defined according to the frequency of semi-permanent nail polish use, using non-paired, non-parametric tests (Kruskal–Wallis), followed by post hoc multiple comparisons.

Within each group, comparisons between samples without and with cosmetic nail varnish were performed using non-parametric paired *t*-tests (Wilcoxon matched-pair signed rank tests). In addition, the comparison of DNA quantity (autosomal and Y chromosome) and degradation index between samples without and with cosmetic nail varnish was repeated using Wilcoxon matched-pair signed rank tests, considering the combined B + C groups, which were merged in a single group. Statistical analyses and figures were created with Prism (v 10.2.1, GraphPad software, LLC, Boston, MA, USA), setting *p* < 0.05 for significance.

The interpretation of electrophoretic data was carried out according to the national Ge.F.I. recommendations [21]. The generated DNA profiles were classified into full or partial profiles, showing a number of correctly typed loci less than that required by the multiplex PCR panel, and single-source profiles, with ≥10 STR loci successfully amplified and characterised by no more than two alleles at each locus, or mixed profiles, with ≥10 STR loci successfully amplified with more than two alleles detected on at least two different loci. To complete the profile outcomes, mixed profiles were then classified as mixed profiles with a major contributor, when one or two alleles at each locus were in a ratio of peak height  ≥  3:1 relative to the other alleles of the same locus, or mixed profiles with no major contributor, when the allele peak height ratio was <3:1.

DNA profiles were compared with the reference samples from the donors and biostatistical evaluation for likelihood ratio (LR) assessment was performed using LRmix Studio software v. 2.1.5 for single-source, mixed profiles with a major contributor matching the reference and mixed profiles without a major contributor. Values of LR ≥ 10^6^ were considered informative for donor identification. This threshold was chosen because it provides extremely strong support for the prosecution proposition (Hp) rather than the alternative defence proposition (Hd) [22,23].

The resulting genotype data were uploaded to the HIrisPlex-S DNA Phenotyping web tool (https://hirisplex.erasmusmc.nl/, accessed on 7 March 2019) to predict eye, hair, and skin colour. The eye colour probabilities were interpreted using a 0.7 threshold, following Walsh et al. [24], while hair colour predictions were interpreted according to the scheme described in Walsh et al. [25]. For eye colour evaluation, the model proposed by Pośpiech et al. [26] was additionally applied. Skin colour predictions are based on the dermatological Fitzpatrick scale for skin colour and sun sensitivity [19].

## 3. Results

DNA was successfully extracted from all samples, yielding quantities ranging from 0.13 to 21.5 ng/μL and from 0.01 to 5.4 ng/μL, respectively, for fingernails without and with semi-permanent nail polish. The aliquot of the cosmetic products, analysed as a negative control, did not show quantifiable DNA. Male DNA was detected in 73.8% (*n* = 31/42) of the samples, consisting of 18 samples without (86%) and 13 samples with (62%) semi-permanent nail polish (Table 2). The [AUTO]/[Y] ratios remained well above 2, ranging from 77 to 13,377. In one of the 42 samples, the male DNA quantity was comparable to the autosomal DNA ([AUTO]:0.75 ng/μL; [Y]:0.62 ng/μL). Therefore, this volunteer was excluded from statistical analyses. The PowerQuant System confirmed the absence of inhibitors in all tested samples. Autosomal and Y chromosome DNA concentrations, as well as degradation index ranges, are shown in Table 2. More details can be found in the Supplementary Material (S1).

**Figure 3:**
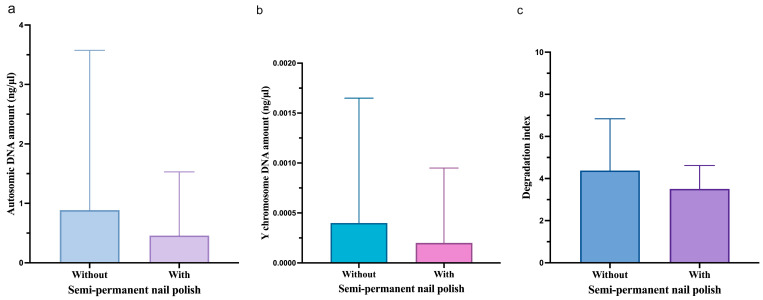
Autosomal (**a**), Y chromosome DNA concentration (**b**), and degradation index (**c**) shown as median and IQR, for fingernails without and with semi-permanent nail polish.

No statistically significant difference was observed between fingerprints without and with cosmetic nail varnish for either autosomal or Y chromosome DNA quantity or degradation index, as shown in Figure 3.

**Figure 4:**
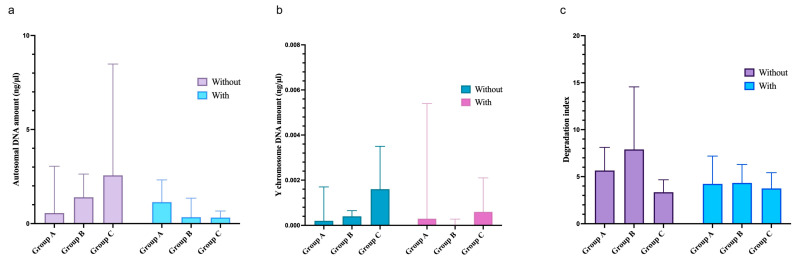
Median and interquartile range of autosomal (**a**) and Y chromosome DNA quantity (**b**), and degradation index (**c**) comparing the three groups (A, frequent users; B, occasional users; C, new users) for fingernails with and without semi-permanent nail polish.

Considering the three groups of semi-permanent nail polish frequency of use, Groups A, B and C were compared among them, showing no statistically significant differences in autosomal (Figure 4a), Y chromosome DNA quantity (Figure 4b) or degradation index (Figure 4c).

Within each group, comparisons between samples without and with cosmetic nail varnish were also performed, revealing no statistically significant differences in autosomal and Y chromosome DNA or in degradation index (Figure 4).

**Figure 5:**
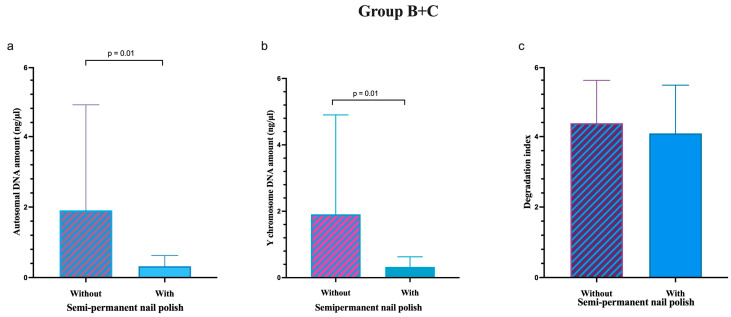
Median and interquartile range of autosomal DNA quantity (**a**), Y chromosome DNA quantity (**b**), and degradation index (**c**) combining Groups B and C for fingernails without and with semi-permanent nail polish.

When Groups B and C were analysed together, a statistically significant difference was observed in both autosomal and Y chromosome DNA recovery between samples without and with cosmetic nail varnish, with *p* = 0.01 for both (Figure 5). In contrast, the degradation index was not significantly different between unvarnished and varnished samples in the combined Group B + C group.

STR typing analysis yielded 39 (93%) full DNA profiles from the 42 samples analysed, while 3 samples (7%) showed partial profiles with allelic drop-out, ranging from 1 to 7 STR loci, and locus drop-out, ranging from 1 to 5 STR loci. No profiles were classified as inconclusive. No differences in IQC markers were observed between polished and unpolished samples. STR studied parameters data are reported in Supplementary Material (S2). Table 3 includes the number of samples amplified from nails without and with semi-permanent nail polish and the DNA profiles obtained. In the majority of samples (*n* = 30/42), single-source profiles were obtained from donor fingernails, both in the absence and in the presence of semi-permanent nail polish.

DNA mixtures were observed in 12 samples (28.6%) with a maximum of alleles/locus  ≤  4, 5 generated from fingernails without cosmetic nail varnish, all showing a major contributor, and 7 from fingernails with cosmetic nail varnish, 6 of which had a major contributor, and 1 showed no major contributor for 3 loci. Of the 11 mixed profiles with a major contributor, 10 matched the reference donor, including 4 samples without and 6 samples with semi-permanent nail polish (Table 3). The only mixed profile with a major contributor not matching the volunteer was found in a fingernail sample without nail polish. The minor contributor showed all the donor’s alleles, but we did not perform the LR calculation of this component.

A Y chromosome peak was observed in 6 genetic mixed profiles of which 3 were from fingernails without nail polish, showing in one of these a major contributor not matching the volunteer.

The biostatistical calculation was performed on both single-source profiles, mixed profiles with major contributor matching the volunteer and for the only mixed profile without a major contributor resulting the LR value in a range of 10^24^  <  LR  <  10^32^.

Only 6 of the 21 analysed samples were compliant with the criteria established for FDP analysis, specifically showing a DNA quantity ranging between 0.46 and 5.3 ng/μL no detectable male DNA, and a single-source genetic profile. FDP analysis showed successful processing of all libraries, with an average coverage of 2031.8 and mean coverage uniformity of 97.8%. Complete genotype profiles were obtained for all 41 phenotype-informative DNA markers in every sample, according to the applied coverage thresholds. Predictions obtained from the HIrisPlex-S web tool were compared with participants’ self-reported phenotypes, revealing an overall concordance of 72% with the phenotypic categories. Specifically, four individuals self-reported brown eye colour and one individual self-reported blue eye colour; predictions for these phenotypes were accurate in 100% of cases. In contrast, one individual with an intermediate eye colour was incorrectly predicted as blue, resulting in no correct prediction for this category. In the analysed sample, the genotypes CC at rs12913832 and GG at rs1800407 were observed. Applied to the model by Pośpiech et al. [26], this genotypic combination was associated with an increased probability of green eye colour, allowing the correct prediction of the green/blue phenotype. As for hair colour, a comparison between predicted and self-reported data revealed four matches (67%) and two discrepancies (33%). The incorrect predictions involved two individuals reporting brown and dark-brown hair: in one case, a darker hair colour than reported was predicted and in the other case a lighter shade (blond/dark blond). Skin colour prediction showed an accuracy of 67%, with four volunteers classified in the intermediate category (Fitzpatrick III/IV). One discrepant case and one inconclusive case were also observed. The latter exhibited prediction probabilities that fell below the established threshold, and was consequently classified as N.A.

## 4. Discussion

In our study, we analysed nails varnished with semi-permanent polish as a source of DNA for identification purposes. Actually, nails can provide valuable forensic information, especially in cases of degraded human remains for identification of cadavers or kinship analysis as a tool for direct or reverse parentage testing. They are easy to collect, are well-preserved even after sampling, and can be obtained with minimal visible damage to valuable specimens [10,27].

In this study, we evaluated whether the quality and quantity of nuclear DNA could be affected by degradation or inhibition to interfere with DNA profiling when nails were treated in experimental conditions in 21 volunteers subdivided in three groups based on their habit of nail beautification (A, B, and C).

To assess the effect of semi-permanent nail polish, a comparative analysis was firstly conducted between samples without and with semi-permanent nail polish, considering all volunteers. The results showed no significant difference in quantifiable autosomal and Y chromosome human DNA in all comparisons, as well as in degradation index, suggesting that semi-permanent nail polish application does not negatively affect the suitability of fingernails for STR profiling. Notably, the nails with and without semi-permanent nail polish showed high inter-individual variability in the amount of DNA recovered, particularly in the unpolished group; a similar trend was observed for the degradation index. This variability may be attributed to intrinsic characteristics of the donor’s nails, such as cell density and keratinisation processes, which vary not only among individuals within a species but also within the same individual over time [28].

The DNA amount recovered from nails with and without nail polish was compared both among A, B and C groups and within each group, showing no significant differences in any of these comparisons. Because DNA recovery did not differ significantly between groups B and C, which represent volunteers with occasional and regular exposure to aesthetic treatments, these two groups were combined for subsequent statistical analyses. In the combined B + C group, comparison between varnished and unvarnished samples revealed a significant lower DNA yield from nails with semi-permanent polish. This suggests that pooling samples from volunteers with repeated exposure increased the statistical power to detect differences associated with the presence of semi-permanent nail polish.

The observed reduction, although not accompanied by a significant increase in degradation index, may be attributable to repeated mechanical or chemical stress and UV exposure during cosmetic treatments, considering that a recent study demonstrated DNA damage by oxidative stress in cells exposed to frequent UV radiation [17].

Nevertheless, the recovered quantities were suitable to successfully perform STR profiling. Indeed, considering all 42 fingernail samples analysed, only three samples generated partial profiles, with a minimum of 18 loci plus amelogenin, allowing a comparison with the corresponding reference profiles, following the national recommendations [21].

Fingernail samples with and without semi-permanent nail polish predominantly generated single-source profiles in 71.4% of samples, and in mixed profiles, the major contributor corresponded to the volunteer in 91% of cases. This result confirms that semi-permanent nail polish does not affect personal identification. LR values were always over > 10^24^, even in the mixed profile obtained from one varnished fingernail sample that showed no major contributor.

Mixed STR profiles, observed in 28.6% of the samples, indicated that the nail washing procedure employed before DNA extraction, a mild decontamination protocol used to preserve endogenous DNA yield [11], did not completely remove exogenous DNA. Particularly, male DNA was detected in 73.8% of samples even if the recovered amounts of Y DNA were quantified in a few picograms. Indeed, a Y chromosome peak at the amelogenin locus was observed in only six samples. Moreover, in one mixed profile, derived from a fingernail sample without nail polish, with a male DNA quantity similar to the autosomal one, the major contributor did not match the volunteer; her alleles were, however, present in the minor component of the profile.

As exogenous DNA can complicate the interpretation of mixture profiles [29,30,31], future studies should consider optimised decontamination strategies for personal identification, such as brief sodium hypochlorite treatments or lysis buffer soaks [27,32]. Alternatively, toenails may represent a suitable source for identification purposes in degraded bodies, considering their lower exposure to external contamination [33]. On the other hand, a mild decontamination strategy, such as the one employed in our study, might preserve foreign DNA, providing additional investigative information in criminal cases.

The presence of mixture profiles could also hamper the application of FDP analysis. Actually, to evaluate the feasibility of FDP analysis on varnished fingernails, only six samples were selected, excluding mixtures and low DNA amounts. The small number of samples could be a limitation of this study, but phenotypic predictions for eye, hair, and skin colour were obtained for all analysed samples, and the observed discrepancies were not associated with the presence of nail polish, supporting the feasibility of FDP analysis also from these samples.

Incorrect predictions were mainly attributable to known limitations of the HIrisPlex-S model, as reported in the literature [34], particularly for complex phenotypic traits such as intermediate eye colour or variable hair shades. Indeed, one discrepancy in eye colour was clarified by integrating the model by Pośpiech et al. [26], while hair colour discrepancies may have resulted from progressive hair darkening during childhood [25].

Taken together, the results of the study suggest that, while the use of semi-permanent nail polish, particularly when applied repeatedly, may result in a reduction in the amount of DNA recovered, STR typing from treated fingernails remains a viable and informative method for individual identification. Nevertheless, the potential presence of exogenous DNA highlights the need for cautious interpretation of mixed STR profiles obtained from nail samples, particularly when the pre-wash removal of the foreign DNA is not effective. These findings emphasise the importance of the decontamination step on nails for the donor identification purposes. However, the specific investigative context could require the analysis of the foreign DNA eventually deposited on nails.

Finally, the application of FDP analysis on semi-permanent nail polish is feasible but limited by DNA mixture detection and dependant on the quantity of DNA.

## 5. Conclusions

In this study, we report the usefulness of DNA analysis from fingernails that have been treated with semi-permanent nail varnish for forensic DNA profiling and phenotyping. Forensic DNA analysis must consider how the evolution in societal habits, including cosmetic treatments, may influence the availability and quality of biological evidence found at crime scenes. In this context, fingernails treated with semi-permanent nail polish—a cosmetic practice that has become increasingly widespread in recent years—should still be regarded as a valuable alternative source of DNA in forensic analyses involving degraded human remains.

## Figures and Tables

**Table 1:** Products used for the application of semi-permanent nail polish, including ingredients and curing times for each applied layer. * = Air-dried, no UV/LED curing.

Products	Ingredients	Curing Times
Nail prep	Isopropyl Alcohol, Ethyl Acetate, Isopropyl Acetate	30 s *
Acid-free primer	ethyl acetate, bis-HEMA poly(1,4-butanediol)-9/IPDI copolymer, isobornyl acrylate, hydroxypropyl methacrylate	30 s *
Base gel defence coat	2-hydroxyethyl methacrylate (HEMA), methacryloylethyl phosphate, trimethylolpropane triacrylate, ethyl trimethylbenzoyl phenylphosphinate, BHT	30 s
Gel polish colour	di-HEMA trimethylhexyl dicarbamate, HEMA, hydroxypropyl methacrylate, PEG-9 dimethacrylate, isopropyl alcohol, butyl acetate, ethyl acetate, hydroxycyclohexyl phenyl ketone, trimethylbenzoyl diphenylphosphine oxide	60 s
Top coat	4-hydroxybutyl acrylate, di-HEMA trimethylhexyl dicarbamate, hydroxypropyl methacrylate, acryloyl morpholine, trimethylolpropane trimethacrylate, ethyl trimethylbenzoyl phenylphosphinate, hydroquinone	90 s

**Table 2:** Summary of the DNA quantification and degradation index results for fingernail samples without and with semi-permanent nail polish. The DNA quantification include autosomal and male DNA concentrations (ng/µL) and is reported as mean ± standard deviation (SD) and as median with interquartile range (IQR). The degradation index is shown as the observed range of values exceeding 2, n. = number of samples.

Fingernails	Autosomal DNA ng/μL		Male DNA ng/μL		Degradation Index	
	Mean and SD	Median and (IQR)	Mean and SD	n. Samples	Range (>2)	n. Samples
without semi-permanent nail polish	2.96 ± 4.8	0.89 (0.52–3.57)	0.03 ± 0.13	18 (86%)	3–21	14 (67%)
with semi-permanent nail polish	1.14 ± 1.5	0.46 (0.17–1.53)	0.001 ± 0.001	13 (62%)	3–11	13 (62%)

**Table 3:** Genotyping results for fingernail samples without and with semi-permanent nail polish. Number (n.) of successfully amplified samples, classified into single-source profiles and mixed profiles, further subdivided according to the absence or presence of a major contributor.

Fingernails	n. Samples Amplified	Full Profile	Partial Profile	Single-Source Profiles	Mixed Profile	
					Without Major Contributor	With Major Contributor
without semi-permanent nail polish	21	21	0	16 (76%)	-	5 (24%)–4/5 matching the volunteer
with semi-permanent nail polish	21	18	3	14 (67%)	1 (5%)	6 (28%)–6/6 matching the volunteer

## Data Availability

The original contributions presented in this study are included in the article/Supplementary Materials. Further inquiries can be directed to the corresponding author.

## Supplementary Materials

The following supporting information can be downloaded at: https://www.mdpi.com/article/10.3390/genes17030322/s1. S1_Quantification data: Table S1: DNA quantification results; S2_STR Data_studied parameters: Table S2: PCR amplification and interpretation parameters applied for STR analysis; Table S3: Number (n.) of profiles showing peak imbalance; Table S4: Number (n.) of samples showing peak imbalance per STR marker.
